# Woman with Three Breasts

**Published:** 1865-02

**Authors:** 


*Woman with Three Breasts.*—Dr. Schofield Johnson reports the case of a woman, whom he attended in her second confinement, having three breasts—two well formed, and somewhat larger than usual. Immediately below the left mamma was a supernumerary breast about two inches and a half in diameter, full of milk, and with a well developed areola and nipple. When not suckling, this nipple resembles a mole, and lies flat, for which it was taken until pregnancy revealed its true character.—*Lancet.*

